# Physiotherapeutic interventions on quadriceps muscle architecture in patello-femoral pain syndrome

**DOI:** 10.6026/97320630019454

**Published:** 2023-04-30

**Authors:** Vinaya Kumar MV, Navin Bala Subramanian, Sreelatha S, Sai Kotamraju, Madhan Krishnan

**Affiliations:** 1 Department of Anatomy, MallaReddy Medical College for Women, Suraram, Hyderabad- 500055, Telangana, India; 2 Department of Sports Medicine, Saveetha Medical College and Hospital, SIMATS, Thandalam- 602105, Tamilnadu, India; 3 Department of Radiology, MallaReddy Medical College for Women, Suraram, Hyderabad- 500055, Telangana, India; 4 Faculty of Allied Health Sciences, Chettinad Hospital and Research Institute, Chettinad Academy of Research and Education, Kelambakkam-603103, Tamilnadu, India

**Keywords:** Physiotherapeutic intervention, Quadriceps Muscle, patellofemoral pain syndrome

## Abstract

Quadriceps weakness and morphological alteration is a documented phenomenon that can have a major impact on strength and functional performance of PFPS patients. An effective and trustworthy non-invasive technique for measuring the quadriceps muscle's anatomy and architecture is B- Mode Ultrasonography. The aim of the study is to assess and compare the effectiveness of neuromuscular electrical stimulation application, quadriceps strengthening and in combination on the quadriceps muscle architecture and functional capacity in patients with Patello femoral pain syndrome. One hundred and twenty-four participants aged 18 - 40 years old with anterior knee pain were included. Participants were randomly allocated into four groups. Group A participants were given NMES, group B were given QS, group C were given combination therapy (NMES+QS) and group D was control group. Cross sectional area, Fascicle length and pennation angle were measured using B-Mode (2D) ultrasound for Quadriceps Femoris muscle. Knee function and pain were assessed using Kujala score and VAS. All parameters were evaluated before and after the intervention. The mean age, weight, height and BMI of control, NMES, QS and NMES+QS were not statistically significant (P = 0.881, 0.960, 0.951 and 0.953) which shows that the control and experimental groups were homogenous. Combination group showed significant improvement when compared to QS group followed by NMES group. Control group did not show any improvement. Neuromuscular Electrical Stimulation in combination with quadriceps strengthening showed a better outcome than in isolation on quadriceps muscle architecture after 10 weeks.

## Background:

Patello femoral pain is the common musculoskeletal disorder which is also referred as anterior knee discomfort, runner's knee, patellofemoral pain syndrome, and chondromalacia patellae. It is characterized by pain behind or around the patella. In general practice, 11-17% of all knee pain cases involve patellofemoral discomfort, which is widespread. It affects people of all ages and activity levels [1]. Women affects two times more than men [2]. Typically, patients report generalised anterior knee pain that is made worse by daily activities that load flexed knees, such as squatting, climbing stairs, and running.Patellofemoral syndromes etiologic are unclear, but it is mostly multifactorial and related to training methods. Six anatomical regions, including the synovium, subchondral bone, skin, retinaculum, muscle and nerve are thought to be involved. According to studies, four main causes include trauma, overactivity/overload, lower extremity muscle imbalance, and malalignment in the lower extremities and/or patella. The fourth contributing element, excessive use, seems to be the most crucial. Moreover, compared to athletes who participate in multiple sports, early sport specialisation practises have been demonstrated to 1.5 fold increase the relative risk of PFPS [3]. Skeletal muscle quality and architecture are closely related to muscle strength. The thickness, cross-sectional area (CSA), fascicle length (FL) and pennation angle or fibre angle (PA) of a muscle are important factors in the creation of force and the operation of the muscle during bodily motions. A typical side effect of knee diseases is quadriceps weakness, which can be brought on by both muscle atrophy and neural inhibition, which inhibits the muscle from fully activating [4]. Quadriceps atrophy and morphological alteration is a documented phenomenon that can have a major impact on strength and functional performance of PFPS patients. An effective and trustworthy non-invasive technique for measuring the quadriceps muscle's anatomy and architecture is real-time ultrasound. Anatomical architectural properties of the quadriceps muscle group [Vastus medialis (VM), vastus intermedius (VI) , vastus lateralis (VL) and rectus femoris (RF)] such as cross sectional area, fascicle length and pennation angle are quantified using RTUS [5]. Although both operative and non-operative methods are utilised to treat PFPS, and many patients get better with non-operative therapy. As PFPS is a non-degenerative disease, conservative treatment frequently produces full recovery, especially in young patients [6, 7]. Both weight-bearing and non-weight bearing quadriceps strengthening exercises demonstrated increased muscle strength, decreased pain, and increased function on the Kujala scale [8]. Several studies have recommended the use of neuromuscular electrical stimulation (NMES) for the treatment of PFPS [9]. Therefore, it is of interest to evaluate the effectiveness of physiotherapeutic interventions among subjects with patellofemoral pain syndrome and to identify the improvement in architectural parameters of quadriceps muscle, as a whole or any individual part, using ultrasonic imaging technique for individuals with PFPS and comparing this to a contralateral, asymptomatic limb.

## Methods:

## Study design:

A randomized controlled study was conducted to evaluate the effectiveness of Neuromuscular stimulation (NMES), Quadriceps strengthening (QS) or in combination of both (NMES+QS) on quadriceps muscle architecture, Q angle, Knee function and pain intensity among patients with Patellofemoral pain syndrome.These parameters were measured in four groups of subjects; NMES group (group A) underwent electrical stimulation, QS group (group B) underwent quadriceps strengthening , Combination group (group C) and control group (group D) who were given sham therapy with very low intensity NMES. All subjects offour groups were tested at baseline (0 week) and endline (10 weeks) intervention period.

## Sampling technique:

Purposive sampling has been adopted to identify the subjects in the study. Random sampling technique was adopted to allocate the subjects for intervention. The lottery method was used for the randomization process. Researcher has prepared opaque, sealed and numbered envelopes containing the modality of treatment. The researcher unzipped the envelope and assigned that intervention to each participant when they signed up for the trial. Double blinding technique was adopted in the study. Radiologist was blinded about the intervention given to the participant and therapist was blinded about the group the subject allocated to.

## Participants:

One hundred and fifty participants including both males and females aged 18-40 years having anterior knee pain were referred by the orthopaedic department. One hundred and twenty four participants completed the study. Participants of the study were recruited from out patients who have attended the department of Orthopaedics for knee pain. Doctors in the orthopaedic department of Malla Reddy Narayana multi-speciality Hospital were informed about the aims and objects of the study and requested them to refer patients with PFPS. Participants were informed about the study and their informed consent was obtained. Study was conducted with the approval of MallaReddy Medical College for Women institutional ethical committee (Approval number MRMCWIEC/AP/75/2021). Subjects are included in the study if they had pain for at least one month around or behind the patella while doing two or more of the following activities such as standing, sitting down after a lengthy period of standing, kneeling, squatting, climbing, or descending stairs, or running. Patients who have had ligament, meniscus, or bone injuries, as well as those who have referred pain from lesions of the lumbar spine, hip, or ankle, or who have neuromuscular problems, are not eligible for the study. Anyone who is unwilling was excluded from the study. If the participants have bilateral knee pain the most symptomatic knee was taken into consideration.

## Assessment:

The subjects were evaluated two times: baseline assessment at 0 week and end line evaluation at ten weeks after receiving intervention. The both evaluations consisted of a clinical examination, ultrasonic examination, and specific measurements includingKujala score, Visual Analog Scale (VAS), Quadriceps angle (Q angle), Cross sectional area, fascicle length and Pennation angle of Vastus medialis (VM), Vastus intermedius (VI), Vastus lateralis (VL), and Rectus femoris (RF) muscles along with pain measures. These measurements were re-evaluated at the end of the ten weeks, always in the same order. Anthropometrical measurements such as heights and weights were measured by a weight and height scales were done for all subjects. Subjects were weighed in kilograms (kg). Each subject's stature was measured in metres (m). BMI was calculated to all subjects and based on BMI they were categorized into healthy, underweight, overweight and obese.Pain assessment was done using 10- point visual analogue pain scale, with zero representing no pain and ten representing severe agony. VAS was recorded twice, baseline at zero weeks and endline after 10 weeks of intervention. Knee function was assessed by Kujala score. It is an independent questionnaire designed to evaluate the severity of symptoms and physical limitations in PFPS patients. 13 questions on specific activities, pain intensity, and clinical symptoms make up the self-administered questionnaire for PFPS patients. Q angle was measured in degrees using goniometer.

##  Muscle architecture:

Muscle measurements of the affected knee were examined in vivo at rest, using a 2-D B- mode ultrasonography (EPIQ ELITE, Philips )with a lineararray transducer of 10-15MHZto assess cross sectional area, pennation angles, and fascicle lengths. Ultrasonography was done on vastus medialis (VM), vastus intermedius (VI), vastus lateralis (VL) and rectus femoris (RF). For the photographs, subjects were lying supine with their legs flexedat 10 degrees and their muscles relaxed. All measures were made after the participant had been in the supine position for at least 10 minutes to allow for fluid shift. In order to facilitate acoustic coupling, a water-soluble gel was then placed on the device's transducer. Cross sectional area(cm2), fascicle length(mm)and pennation angles(0) of vastus medialis (VM), vastus intermedius (VI), vastus lateralis (VL) and rectus femoris (RF) were measured.The distance between the superficial and deep aponeurosis along the fascicular route was used to define fascicle length. The fascicles typically extend beyond the captured image. The missing piece's length was calculated using linearextrapolation. The linear distance between the recognisable end of a fascicle and the point where a line drawn from the fascicle and a line drawn from the superficial aponeurosis connect was measured to achieve this. The angle between the fascicular route and the deep aponeurosis of the muscle was described as the pennation angle. The area of the muscle's cross section which is perpendicular to its fibres and is typically found at the muscle's greatest point is considered as cross-sectional area [10].

## Interventions:

## Neuromuscular Electrical stimulation:

A neuromuscular electrical stimulator was connected to two isolated cables, each connected to a pair of electrodes applied over the motor points of the VL and VM. A biphasic pulsed current was used Frequency (Hz) is the number of pulses in one second (20-50 pulses per second) [8].

[1] Pulse Duration (microsecond) for small muscles is approximately 150-200 and for large muscles 200-300.

[2] Ramp time is at least 2 seconds

[3] ON: OFF time ratio should be set in a way where off time is three times the on time

[4] Treatment time should be between 20 and 30 minutes The frequency of the sessions should be three times a week.

## Procedure:

Carbon-rubber electrodes which are normally coupled to the skin by electrical conductive gel is used. The bipolar electrode placement involves placing both electrodes on the muscle belly or one at the proximal end and another on the distal end of the muscle. During electrical stimulation, it's important to increase the intensity of the stimulation gradually and to the maximum tolerable extent by the patient. For innervated muscles normally, the shorter the pulse duration, the greater the pulse amplitude should be whereas for denervated muscles, both pulse duration and pulse amplitude should be greater than that of innervated muscles which is particularly important to ensure stimulation and sudden contraction of the muscle.

## Quadriceps strengthening:

Participants of the group -B instructed by physiotherapist about the quadriceps strengthening exercises and participants had to perform

[1] Isometric q cep's exercise - 3 sets of 10 repetitions (5 s).

[2] Straight leg raise with 3 sets - 20 repetitions (increasing ankle weights)

[3] Short arc knee extension exercise - 3 sets of 20 repetitions.

The entire exercise regimen is done 3 times a week for 10 weeks.

## Statistical analysis:

Statistical analyses of the data were carried out in Sigma Plot 14.5 version (Systat Software Inc., San Jose, USA). A p-value less than <0.05 was considered to be statistically significant.

## Results:

Mean and standard error of age, weight, height and body mass index (BMI) were given in Table 1. The mean age, weight, height and BMI of control, NMES, QS and NMES+QS were not statistically significant (P = 0.881, 0.960, 0.951 and0.953).This shows that the control and experimental groups were homogenous with respect to age, weight, height and BMI. Two-way Repeated measures ANOVA was performed to compare the average Kujala score, Visual Analog scale and Q angle between different intervention and control groups and also over the period of time. More details are provided in the Table 2.

The data are represented as mean + SEM and analysed by two-way repeated measures analysis of variance (RM ANOVA) for one factor repetition, and Bonferroni 't' test for post-hoc multiple comparisons. Factor A, was groups (between group comparison - Control, NES, QS and NES+QS), Factor B, was tests (within group comparison i.e., repetition factor - Pre-test and Post-test) and the group X test interaction. A probability of 0.05 and less was considered as statistically significant. Two-way RM ANOVA revealed statistical significance for groups, tests and group X test interaction (P < 0.001, < 0.001 and < 0.001, respectively) for kujala score, but did not show statistical significance for groups for both VAS and Q angle (P= 0.672 and 0.824).From the pre-test to post-test the control group did not show any improvement in kujala score, VAS and Q angle. The order of improvement was Controlandlt;NMESandlt;QSandlt; Combination NMES+QS, and the combination group NMES+QS was the best in improving Kujala score, VAS andQ angle. Details are depicted in Table 3.

Two-way RM ANOVA revealed statistical significance for tests and group X test interaction (P < 0.001 and < 0.001 respectively) but did not show statistical significance for groups with respect to CSA, FL and PA of vastus medialis, FL and PA of vastus intermedius, vastus lateralis and Rectus femoris. It showed statistical significance for groups with respect to CSA of VI, VL and RF (P < 0.001, P = 0.003 and 0.005 respectively). Compared to the control pre-test (between groups), NMES pre-test, QS pre-test and Combination (NMES+QS) pre-test of CSA, FL and PA of VM, VI, VL and RF did not show significance (P = 1.0, 1.0 and 1.0 respectively) except pennation angle of vastus lateralis (P = 0.881). Compared to the control post-test (between groups), QS post-test and Combination (NMES+QS) post-test showed statistical significance for CSA and PA of VM (P = 0.013 and 0.007 and P = 0.019 and 0.009) respectively. Compared to the control post-test (between groups), NMES post-test, QS post-test and Combination (NMES+QS) post test showed significance for CSA of VI ( P< 0.001, < 0.001 and < 0.001 respectively). Compared to the control post-test (between groups), Combination (NMES+QS) post-test showed statistical significance for PA of VI (P = 0.041).Compared to the control post-test (between groups), QS post-test and Combination (NMES+QS) post test showed significance (P< 0.001and < 0.001 respectively) for CSA of VL. Compared to the control post-test (between groups), Combination (NMES+QS) post test showed significance (P< 0.001) for PA of VL. Compared to the control post-test (between groups), Combination (NMES+QS) post test showed significance for CSA and PA of RF (P< 0.001 andP = 0.023 respectively).From the pre-test to post-test the control group did not show any improvement in CSA, FL, PA’s of VM, VI, VL and RF. Compared to NMES pre-test, NMES post-test of VM (CSA, FL andPA), VI (CSA and PA), VL (CSA and PA) and RF (CSA and PA) showed statistical significance (P < 0.001) respectively except fascicle length of VL which showed statistical significance with P = 0.022.However, fascicle lengths of VI and RF did not show significance (P = 0.178and 0.913). These details are depicted in Table 4, Table 5,Table 6Table 7.The order of improvement was Control<NMES<QS< Combination NMES+QS, and the combination group NMES+QS was the best in improving CSA, FL and PA's of VM, VI, VL and RF muscles.

## Discussion:

In the current study, participants with PFPS were examined to determine the in vivo effects of NMES, QS and Combination therapy on quadriceps muscle architecture using ultrasound along with the pain scoreand knee function. The findings of the study showed that muscle ultrasound is a highly reproducible approach for measuring the muscle architecture parameters for all quadriceps muscles which is also suggested by Whittaker *et al* [11]. Our study findings have also proved that receiving physiotherapeutic interventions can help PFPS patients with their pain and knee function along with the improvement in muscle architecture parameters and properties. Whether the individuals received NMES, QS or combination therapy, improvement still occurred. The present study's results fully support the theory since, when compared to the control group, as the intervention group's participants showed substantial improvements in post-test muscle architecture with respect to CSA, FL and PA of VM, VI, VL and RF muscles. One of the factors contributing to the improvement in parameters was the individuals' high rates of compliance to the physiotherapeutic sessions, at 89%, 86%, 89%and 91%, respectively. It was predicted that combination therapy would significantly improve muscle architecture, pain score and knee function. Although we found improvements in muscle architecture parameters in three experimental groups (NMES group, QS group and Combination group (NMES+QS), the order of improvement was Combination group (NMES+QS)>QS group> NMES group [12]. Our findings have a significant therapeutic relevance because, to our knowledge, no similar study has been conducted to investigate all four muscles of the quadriceps in PFPS patients using non-invasive procedure, ultrasonography with different physiotherapeutic interventions.

The measurements of the muscular architecture acquired in this investigation are in good agreement with measurements of the quadriceps muscle assessed in earlier studies using ultrasonography [4]. The mean CSA of VM for the group A was (15.26+2.33), group B was (15.98+2.55), group C was (16.09+2.8) and group D was (13.94+2.7) which is coinciding with Minnehan *et al* [13]. There was significant inverse correlation between CSA of VM and age and positive correlation with BMI. The mean CSA of VI for the group A was (11.62+0.89), group B was (12.02+1.0), group C was (12.47+0.84) and group D was (10.59+0.96). To our knowledge, no other study has reported the muscle architecture of vastus intermedius alone. The mean CSA of VL for the group A was (5.00+0.97), group B was (5.99+0.98), group C was (5.86+1.05) and group D was (4.59+1.01).Reeves *et al* reported the range of the VL muscle CSA as 7.45-12.27 cm2 is slightly greater than our findings [14]. Study conducted by Lixandrao *et al* got very high CSA (21.25 ± 6.85 cm2) [15]. The variation in the CSA might be due to the built of the subjects. The mean CSA of RF for the group A was (2.87+0.47), group B was (2.87+0.51), group C was (3.41+0.55) and group D was (2.72+0.43).Similar findings were presented by El-Ansary *et al* [5]. Two way repeated measures ANOVA indicates significant differences in CSA of all muscles between control and experimental groups (p<0.001).

The mean FL of VM for the group A was (63.7+5.82), group B was (66.23+6.71), group C was (66.90+6.63) and group D was (63.16+6.04). The mean FL of VI for the group A was (57.37+2.84), group B was (57.84+2.82), group C was (58.32+3.08) and group D was (56.93+3.14). The mean FL of VL for the group A was (57.39+3.76), group B was (58.37+3.70), group C was (59.27+4.20) and group D was (56.73+3.52) .The mean FL of RF for the group A was (52.59+3.35), group B was (53.20+3.17), group C was (53.48+3.90) and group D was (51.92+4.22). The variation in fascicle lengths is due to the limited field of view of conventional B-mode ultrasound as it does not always capture the entire fascicle length of the muscle [16]. Two-way repeated measures ANOVA indicates significant differences in fascicle lengths of all muscles between control and experimental groups (p<0.001).

The mean PA of VM for the group A was (18.04+3.27), group B was (18.37+3.18), group C was (18.53+2.72) and group D was (16.22+2.51). The pennation angle of VM was comparatively lesser other studies. Jan *et al* also reported that PA in PFPS patients was significantly smaller than healthy adults [17]. The mean PA of VI for the group A was (11.56+2.90), group B was (11.58+2.91), group C was (12.44+2.73) and group D was (10.51+2.62) .The mean PA of VL for the group A was (11.76+3.11), group B was (12.32+2.85), group C was (13.64+2.71) and group D was (10.62+2.57) andthe mean PA of RF for the group A was (9.28+2.20), group B was (9.48+2.15), group C was (9.85+2.28) and group D was (8.32+1.82). Pennation angles of RF and VL of our study were nearly similar to previous studies (RF=10°-17°, VL=11.9°-14.5°) [18, 19, 20]. However, Sekir *et al* found higher pennation angles of RF and VL measured using ultrasound with fully relaxed knee joint in 00 (VL = 16.70 and RF = 14.60) and nearly similar values at 600 (VL = 14.60 and RF = 12.90) [21]. The reason for variation of PA values is due to the location of ultrasound and the knee position. The CSA, FL and PA of VM was higher than VI, VL and RF. There is no much difference in fascicle lengths of VI and VL. The CSA, FL and PA of RF was comparatively lower than other muscles.

Pre and post Kujala scores along with VAS score revealed a considerable decline in functional impairment and pain. The improvement in knee extensor strength, the decrease in discomfort, and the improvement in lateral patella tracking caused by higher levels of VMO activation are thought to be the causes of the improved Kujala score. In their study suggests that a significant correlation between pain and functional impairment [22]. In a study, Callaghan JM *et al*. found that electric muscle stimulation of the quadriceps significantly improved all outcomes (P = 0.05), including Kujala Score, when used to treat patellofemoral discomfort. Steinkamp *et al* provided evidence that patellofemoral joint response forces are reduced when exercising with a closed kinetic chain between 0 and 40 degrees of knee flexion [23]. In order to balance the strength and flexibility of the quadriceps, they suggested that other muscular groups around the knee need to be generally strengthened. This could be the probable reason for improvement seen in QS group when compared with NMES group. Bennet and Stauber *et al*. [24] reported a remarkable improvement in eccentric quadriceps strength and significant pain reduction among PFPS after only two weeks of open chain concentric-eccentric exercises, despite the fact that it might seem unlikely that strength can improve significantly in one to two weeks.

## Conclusion:

The use of linear transducers on curved surfaces and the pressure applied by the operator applies on the muscle tissue can affect the outcomes. Image distortion might have happened even though we took care to keep the transducer in constant contact with the skin and to maintain the same pressure during the whole scan. It was also difficult to capture quality images among obese patients.

## Figures and Tables

**Table 1:** Analysis of baseline physical parameters for homogeneity.

**S.No.**	**Variable**	**Category**	**Control**	**NMES**	**QS**	**NMES+QS**	**Statistics**
1	Age	Mean	29.7	29.9	29.2	28.8	F = 0.222
	(years)	SEM	1.1	1.1	1.2	1	P = 0.881
2	Weight	Mean	67.4	67.6	68	67.1	F = 0.100
	(kg)	SEM	1.2	1	1.3	1.1	P = 0.960
3	Height	Mean	1.607	1.601	1.612	1.613	F = 0.116
	(m)	SEM	0.015	0.016	0.017	0.017	P = 0.951
4	BMI	Mean	26.6	26.9	26.7	26.3	F = 0.112
	(kg/m2)	SEM	0.9	0.7	0.8	0.8	P = 0.953
Sample size - Control = 32; NMES = 31; QS = 30 and NMES+QS = 31; NMES = Neuromuscular electrical stimulation; QS = Quadriceps strengthening; The 'F' and 'P' values are by one way ANOVA.

**Table 2:** Average Kujala score, Visual Analog scale and Q angle between different intervention and control groups

**Outcome**	**Group A**		**Group B**		**Group C**		**Group D**		**Interaction**	
	n=31		n=30		n=31		n=32			
	Mean	SD	Mean	SD	Mean	SD	Mean	SD	F - value	p – value
Pre Kujala score	58.26	6.5	58.93	5.92	58.1	5.02	59.28	6.07	128.97	<0.001*
Post Kujala score	64.35	5.77	68.43	7	73.23	4.86	58.91	5.85		
Pre Visual Analog Scale	7.3	0.86	7.29	0.67	7.61	0.84	7.09	0.78	709.15	<0.001*
Post Visual Analog Scale	6.97	0.93	6.52	0.68	6.36	0.79	7.09	0.78		
Pre Q Angle	16.42	2.07	16.77	2.06	16.72	1.94	16.32	2.05	88.53	<0.001*
Post Q Angle	15.99	2.01	16.18	1.94	15.29	1.88	16.32	2.06		

**Table 3:** Comparison of control and experimental groups on Kujala score (Kujala), Visual Analog Scale (VSA) and Quadriceps angle (Q angle) by two-way RM ANOVA with Bonferroni 't' test.

**S.No.**	**Group**	**Test**	**Kujala score**	**VSA**	**Q angle**
1	Control	Pre-test	59.28 ±1.07	7.10 ± 0.14	16.32 ± 0.36
	NMES	Pre-test	58.26 ± 1.17	7.30 ±0.15	16.42 ±0.37
	QS	Pre-test	58.93 ± 1.08	7.29 ± 0.12	16.78 ± 0.38
	NMES+QS	Pre-test	58.10 ± 0.90	7.62 ± 0.15	16.72 ± 0.35
	Control	Post-test	58.91 ± 1.03	7.10 ± 0.14	16.32 ± 0.36
	NMES	Post-test	64.36 ± 1.04	6.97 ± 0.17	16.00 ± 0.36
	QS	Post-test	68.43 ± 1.28	6.52 ± 0.13	16.18 ± 0.35
	NMES+QS	Post-test	73.23 ± 0.87	6.37 ± 0.14	15.30 ± 0.34

**Table 4:** Comparison of control and experimental groups on Vastus medialis cross sectional area (VM_CSA), Vastus medialis fascicle length (VM_FL) and Vastus medialis pennation angle (VM_PA) by two-way RM ANOVA with Bonferroni 't' test.

**S.No.**	**Group**	**Test**	**VM_CSA**	**VM_FL**	** VM_PA**
1	Control	Pre-test	13.93 ± 0.48	63.20 ±1.06	16.21 ± 0.45
	NMES	Pre-test	13.98 ± 0.41	63.47±1.05	16.88 ± 0.53
	QS	Pre-test	14.18 ± 0.43	64.16±1.21	16.62 ± 0.53
	NMES+QS	Pre-test	13.71 ± 0.47	62.56 ± 1.22	16.41 ± 0.44
	Control	Post-test	13.94 ± 0.48	63.15 ± 1.06	16.21 ± 0.44
	NMES	Post-test	15.26 ± 0.42	63.69 ± 1.05	18.04 ± 0.59
	QS	Post-test	15.98 ± 0.47	66.23 ± 1.22	18.37 ± 0.58
	NMES+QS	Post-test	16.10 ± 0.50	66.89 ± 1.20	18.53 ± 0.49

**Table 5:** Comparison of control and experimental groups on Vastus intermedius cross sectional area (VI_CSA), Vastus intermedius fascicle length (VI_FL) and Vastus intermedius pennation angle (VI_PA) by two-way RM ANOVA with Bonferroni 't' test.

**S.No.**	**Group**	**Test**	**VI_CSA**	**VI_FL**	**VI_PA**
1	Control	Pre-test	10.60± 0.17	56.94±0.55	10.50± 0.47
	NMES	Pre-test	10.78± 0.16	57.28±0.51	11.16± 0.53
	QS	Pre-test	10.90± 0.82	57.35±0.52	11.01± 0.52
	NMES+QS	Pre-test	11.00± 0.16	56.20±0.57	10.97± 0.49
	Control	Post-test	10.59± 0.17	56.93±0.56	10.51 ± 0.46
	NMES	Post-test	11.62± 0.89	57.37±0.51	11.56± 0.52
	QS	Post-test	12.02±1.00	57.84±0.52	11.58± 0.53
	NMES+QS	Post-test	12.47± 0.84	58.32±0.55	12.44± 0.49

**Table 6:** Comparison of control and experimental groups on Vastus lateralis cross sectional area(VL_CSA), Vastus lateralis fascicle length (VI_FL) and Vastus lateralis pennation angle (VI_PA) by two-way RM ANOVA with Bonferroni 't' test.

**S.No.**	**Group**	**Test**	**VL_CSA**	**VL_FL**	** VL_PA**
1	Control	Pre-test	4.584 ± 0.18	56.73 ± 0.62	10.63 ± 0.46
	NMES	Pre-test	4.607 ± 0.18	57.13 ± 0.68	11.23 ± 0.53
	QS	Pre-test	4.832 ± 0.16	56.72 ± 0.68	11.11 ± 0.50
	NMES+QS	Pre-test	4.633 ± 0.18	57.65 ± 0.76	11.64 ± 0.45
	Control	Post-test	4.587 ± 0.18	56.73 ± 0.62	10.61 ± 0.46
	NMES	Post-test	5.000 ± 0.17	57.40 ± 0.68	11.75 ± 0.56
	QS	Post-test	6.000 ± 0.18	58.36 ± 0.68	12.32 ± 0.52
	NMES+QS	Post-test	5.855 ± 0.19	59.27 ± 0.75	13.64 ± 0.49

**Table 7:** Comparison of control and experimental groups on Rectus femoris (RF_CSA), Rectus femoris fascicle length (RF_FL) and Rectus femoris pennation angle (RF_PA) by two-way RM ANOVA with Bonferroni 't' test.

**S.No.**	**Group**	**Test**	**RF_CSA**	**RF_FL**	**RF_PA**
1	Control	Pre-test	2.72 ± 0.08	51.92 ± 0.74	8.32± 0.32
	NMES	Pre-test	2.79 ± 0.08	52.57 ± 0.63	8.65 ± 0.37
	QS	Pre-test	2.74± 0.09	52.18 ± 0.63	8.53 ± 0.37
	NMES+QS	Pre-test	2.84 ± 0.07	50.90 ± 0.73	8.53 ± 0.37
	Control	Post-test	2.72± 0.08	51.92 ± 0.75	8.32 ± 0.32
	NMES	Post-test	2.87± 0.08	52.60 ± 0.60	9.28 ± 0.39
	QS	Post-test	2.87 ± 0.09	53.20 ± 0.58	9.48 ± 0.39
	NMES+QS	Post-test	3.42± 0.10	53.47 ± 0.70	9.85 ± 0.41
